# Robotic Diagnosis and Management of Acute Cholecystocolonic Fistula

**DOI:** 10.7759/cureus.24101

**Published:** 2022-04-13

**Authors:** Rachel M Krzeczowski, Heather M Grossman Verner, Brian Figueroa, Jennifer Burris

**Affiliations:** 1 Clinical Research, Methodist Health System, Dallas, USA; 2 Trauma and Acute Care Surgery, Methodist Health System, Dallas, USA

**Keywords:** cholecystocolonic fistula, cholecystitis, robotic surgical management, laparoscopy, biliary tract disease

## Abstract

Cholecystocolonic fistula (CCF) is a rare complication of biliary tract disease. Increased use of imaging has aided in diagnosing these fistulae preoperatively and has established laparoscopy as a safe alternative to laparotomy. Here, we present a 79-year-old male who presented to the emergency room with abdominal pain and was diagnosed with choledocholithiasis. CT scan revealed a CCF, and he underwent endoscopic retrograde cholangiopancreatography (ERCP). He was followed closely to allow maturation of the fistula, and then, da Vinci® Xi robotic cholecystectomy and ligation were performed. Although current comparisons to laparoscopy have yet to demonstrate a clinical advantage, robotic assistance enhances dexterity, visualization, and ergonomics. Our case is one of the first documented successful operative management of CCF using the da Vinci^®^ Xi robot.

## Introduction

The overall incidence of cholecystoenteric fistula in biliary cases is 0.9%-3.2% [1,2]. Of the cholecystoenteric fistula, 8% are cholecystocolonic (CCF), while the remainder involves the duodenum [2]. Symptoms associated with CCF include abdominal pain, fever, jaundice, cholangitis, pancreatitis, melena, and bile salt diarrhea [1-6]. Patients with CCF may also have malabsorption of and deficiencies in fat-soluble vitamins and potassium [4]. The average duration of illness for patients with CCF is four years [2].

A consensus for the imaging of CCF has not been established. Preoperative detection of CCF occurs in only 7.9% of cases [3]. Ultrasound seldom shows a CCF [7]. However, other imaging modalities can reveal CCF, including magnetic resonance cholangiopancreatogram (MRCP), CT scan, barium enema, endoscopy, and endoscopic retrograde cholangiopancreatography (ERCP) [1,2,4,8].

There is no definitive approach to the management of CCF [3]. In 1995, Ibrahim et al. published the first case report describing the treatment of CCF by laparoscopy [9]. Laparoscopic surgery is now accepted as a safe surgical alternative to laparotomy for the treatment of CCF [3,10]. Stapling the fistula has also been established as a safe technique to treat CCF [6,10]. The evolution from open to minimally invasive surgery is now progressing from laparoscopy to robotic. In 1999, the first robotic-assisted radical prostatectomy was performed in Paris, France, using the da Vinci® Surgical System (Intuitive Surgical, Sunnyvale, CA, USA) [11]. Since then, the da Vinci® robotic technology has progressed to include additional arms, improved ergonomics, compatibility with a single-site approach, and dual console capabilities allowing for surgeon collaboration [11]. The use of the da Vinci® Surgical System has also expanded to gynecologic, general, cardiothoracic, and head and neck surgeries.

We completed one of the first successful treatments of a CCF using the da Vinci® Xi Surgical System. Our case includes multimodality imaging studies, video capture of the operation using the robot, a unique pathology report, and an examination of alternative management strategies.

## Case presentation

The patient is an active 79-year-old male who presented to the emergency department (ED) with crampy abdominal pain that progressed steadily throughout the day. He denied emesis, fever, diarrhea, or prior episodes of similar symptoms. Prior to his visit to the ED, he took magnesium citrate and Pepto-Bismol, but these did not alleviate his symptoms. The patient’s past medical history included hypertension, hyperlipidemia, and carotid stenosis after carotid endarterectomy. There was no past medical history of cholelithiasis, cholecystitis, or colon cancer.

**Figure 1:**
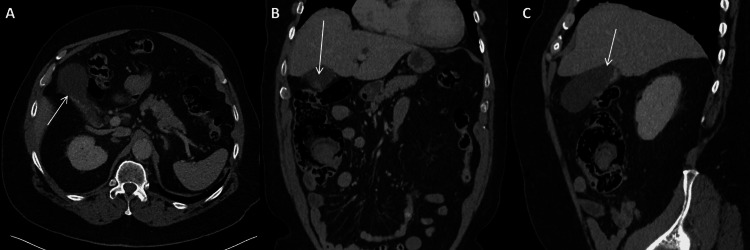
First Preoperative CT This is the first preoperative CT scan. The white arrows identify the gallbladder. Pane A shows the transverse section. Pane B shows the coronal section. Pane C shows the sagittal section.

The patient’s laboratory results included a white blood cell count of 8.1 × 10^3^/µL (normal range: 3.8-10.6 × 10^3^/µL). He had slightly elevated lipase levels (350 U/L) (normal range: 23-300 U/L). At that time, a CT scan (Figure 1A-1C) showed evidence of cholelithiasis, a distended gallbladder, and colonic diverticulosis without signs of inflammation. He had no previous symptoms or diagnosis of diverticulosis. His symptoms resolved after he received pain medication and Phenergan, and he was discharged from the ED.

The patient returned six weeks later with chills and severe, aching pain in the right lower quadrant. He denied nausea, fever, or change in bowel habits. On physical examination, the patient’s abdomen was soft, non-distended, and tender only to deep palpation in the right lower quadrant. Laboratory analysis revealed a total bilirubin level of 3.6 mg/dL (normal range: 0-1.4 mg/dL) and a direct bilirubin level of 2.1 mg/dL (normal range: 0-0.4 mg/dL). Other abnormal values in the chemistry profile included aspartate aminotransferase (AST) of 258 U/L (normal range: 8-42 U/L), alanine aminotransferase (ALT) of 215 U/L (normal range: 13-69 U/L), and alkaline phosphatase of 168 U/L (normal range: 38-126 U/L). He had a critical lipase level of 18,456 U/L (normal range: 23 of 300 U/L). The patient’s white blood cell count was 8 × 10^3^/µL, 77% of which were neutrophils (normal range: 36%-66%).

**Figure 2:**
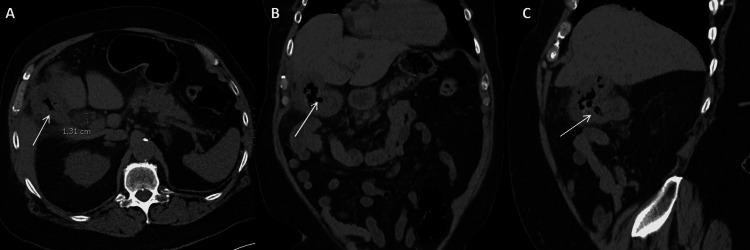
Second Preoperative CT This is the second preoperative CT and was completed six weeks after the first preoperative CT. The white arrows identify the cholecystocolonic fistula. Pane A shows the transverse section with the common bile duct measuring 1.31 cm. Pane B shows the coronal section. Pane C shows the sagittal section.

Imaging (Figure 2A-2C) showed evidence of a dilated common bile duct (13 mm) with surrounding inflammatory changes. Stranding was also noted around the head of the pancreas, as well as the second and third portions of the duodenum. The gallbladder had diffuse wall thickening and emphysema within the lumen, and it appeared to be contiguous with the hepatic flexure of the colon. This was a new finding when compared to the CT scan that was completed six weeks prior.

**Figure 3:**
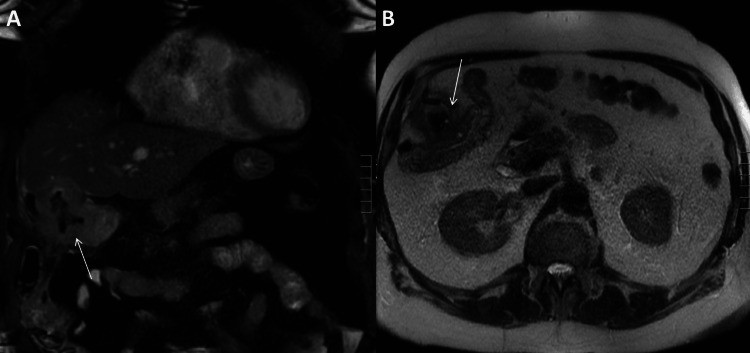
Magnetic Resonance Cholangiopancreatogram This is the preoperative magnetic resonance cholangiopancreatogram. The white arrows identify the cholecystocolonic fistula. Pane A shows the coronal section. Pane B shows the transverse section.

**Figure 4:**
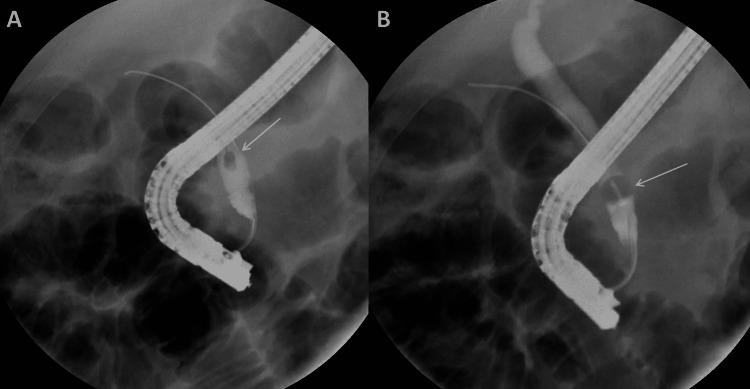
Endoscopic Retrograde Cholangiopancreatography These are the fluoroscopic images from the endoscopic retrograde cholangiopancreatography performed before the fistula takedown operation. The white arrows identify the filling defect in the common bile duct. Pane A shows the side view of the filling defect. Pane B shows the front view of the filling defect.

Diagnoses of both cholecystocolonic fistula and choledocholithiasis were then confirmed by MRCP (Figure 3A, 3B). After consultation with gastroenterology, an ERCP (Figure 4A, 4B) was performed. During the ERCP, a stone was cleared from the common bile duct, and a sphincterotomy was completed. The patient’s symptoms and laboratory values improved after ERCP.

During the same hospitalization, the patient was noted to have atrioventricular nodal reentrant tachycardia, which was ablated by cardiology. Official clearance for surgery was given by cardiology once the ablation was completed. However, given the significant stranding around the pancreas and duodenum, it was decided that delayed intervention would be the safest surgical option. The patient remained on antibiotics to cover colonic bacteria in the event that the colon decompressed into the gallbladder.

The patient was seen in the clinic the following week. He was completely asymptomatic, eating a regular diet, and having normal bowel activity. The original plan was to pursue surgery six weeks after the discovery of the fistula. However, the patient wished to wait longer before undergoing surgery. He completed two additional weeks of antibiotics and was seen in the clinic approximately eight weeks later. At that time, a repeat CT scan demonstrated that the fistula tract was well formed without significant peri-fistula inflammation. The patient had a recent normal colonoscopy, so further evaluation of the colon was not necessary. A preoperative discussion with the patient included an extensive review of the pathophysiology, full disclosure of risks and benefits, and evaluation of all possible management strategies including surgery versus observation. He selected surgery and agreed to proceed with the robotic approach. The robotic approach was selected due to the benefits of improved optics and ease of maneuvering.

Intraoperatively, adhesions were observed extending from the gallbladder to the anterior abdominal wall (Video 1). These were left in place since they provided good retraction of the gallbladder. A window was created posterior to the fistula to reveal the infundibulum of the gallbladder. The gallbladder appeared fully decompressed and narrowed to a cystic duct. The duct and artery were identified. A Kumar Cholangiography Clamp (Nashville Surgical Instruments, Springfield, TN, USA) was employed to perform a perioperative cholangiogram; the hepatic and common bile ducts were easily identified, and contrast flowed into the duodenum. The gallbladder was then removed from the fossa, and the adhesions to the anterior wall were carefully transected. The remaining tissue appeared to be a diminutive gallbladder remnant that was attached to the hepatic flexure. A small abscess cavity and an inflammatory rind were dissected free from the fistulous tract. The tract was divided using the Endo GIA™ stapler (Medtronic, Dublin, Ireland), which was then oversewn and secondarily secured with a small omental flap.

**Video 1:** Cholecystocolonic Fistula Takedown Using the da Vinci Xi Step 1 shows the takedown of adhesions. Step 2 shows the development of the plane between the gallbladder and the colon. Step 3 shows the exposure of the cystic duct and artery. Step 4 shows the intraoperative cholangiogram. Step 5 shows the completion of the cholecystectomy. Step 6 shows the exposure of the fistula. Step 7 shows the ligation of the fistula. The video is narrated by Dr. Jennifer Burris, MD.

Postoperatively, the patient had excellent pain control and was discharged on postoperative day 1. He was seen in the clinic the following week. He described normal energy levels, peroral tolerance, and bowel activity. He had minimal incisional pain and no right upper or right lower quadrant abdominal pain. Blood urea nitrogen and creatinine were minimally elevated at 26 mg/dL (normal range: 10-25 mg/dL) and 1.53 mg/dL (normal range: 0.7-1.4 mg/dL), respectively. Other laboratory tests were normal. The patient exhibited no complications through the six-month follow-up.

The pathologist’s findings were as follows: gallbladder (cholecystectomy), portion of the cystic duct with active cholecystitis; segment of muscular artery, consistent with cystic artery; fibroadipose tissue with fat necrosis; one benign lymph node; no residual gallbladder wall present (the entire specimen was examined microscopically); and no dysplasia or carcinoma identified, and pericolonic fat (excision), fibroadipose tissue with fat necrosis and reactive changes, and no identified malignancy.

## Discussion

Microscopically, the gallbladder was essentially nonexistent, except for the cystic duct and residual fibroadipose tissue. Given the microscopic findings, the pathologist was concerned that the surgeon did not actually remove the gallbladder. However, this finding is not unusual for CCF. Yamashita et al. observed atrophic gallbladder in patients with CCF [2]. Similarly, Gaillard et al. noted intraoperative findings of a shrunken, scarred gallbladder in patients with CCF [1]. In our case, the gallbladder appeared to be fully decompressed into the colon. Additionally, the ERCP with sphincterotomy helped decompress the biliary tree.

CCF is a rare finding that presents with a variety of symptoms, is seldom found preoperatively, and has no definitive management approach. Literature has not reported a specified timeframe for operative intervention for a CCF. In our case, we elected to delay surgery to allow the inflammation to subside. In some cases, delayed treatment is not an option. Often, CCF patients present with emergent complications such as massive bleeding and liver abscess [3]. Fortunately, our patient returned to his baseline once the biliary pancreatitis resolved. The greatest concern with a delayed approach was whether the colonic bacteria would enter the gallbladder and subsequently the biliary tree. Although a CCF ﬁstula is often linked to a high potential for infection of the gallbladder and biliary system, the typical marks of cholangitis are rarely reported as CCF onset symptoms [12]. In our case, the patient was given antibiotics for approximately three weeks. There is no consensus on the length of antibiotic regimens. Because our patient remained completely asymptomatic, we opted to stop antibiotics and continue observation.

Historically, CCF was managed with a two-stage approach that included a diverting colostomy. In the early 1980s, the approach transitioned to one-stage management. In uncomplicated cases, the approach continues to trend toward less aggressive [3]. By delaying our operation, we may have provided a significant reduction in hospital length of stay (LOS) and potentially better overall management. One could argue that, given the lack of air in the gallbladder on follow-up imaging, nonoperative management may have yielded a successful outcome. In patients with significant comorbid conditions, conservative management has afforded an adequate clinical response [4,5].

Until the 1990s, laparoscopy was contraindicated for the treatment of CCFs [13]. Once it was widely accepted, laparoscopy propelled the continuing evolution of surgical technology, including the removal of the gallbladder followed by transection of the fistula using the Endo GIA™ 30 [14,15]. Similarly, CCF were successfully managed with laparoscopy and Endo-GIA™ stapling [6,9]. Laparoscopy has afforded shorter hospital LOS, lower infection rates, improved pain control and cosmesis, and quicker return to the workplace [16]. However, laparoscopy has several limitations, including restricted degrees of motion, 2D video display, and fulcrum-style movements that require the hand to move in the opposite direction of the target [17-19].

Surgical robot development has extended the surgical capabilities of human surgeons beyond the limits of conventional laparoscopy. The 3D camera of the da Vinci® Surgical System evolved from telepresence machines designed for NASA, and it provides optics that are superior to laparoscopic cameras [14,15]. Additionally, the camera is stationary until purposefully moved by the surgeon. The surgeon is effectively provided with a third arm, thus increasing control. Finally, the hinge at the distal tip of the instruments adds back the wrist component lost during laparoscopy. The wrist action enhances suturing ability and improves the learning curve for intracorporeal suturing [16,20]. We found that these developments helped optimize our operative approach.

## Conclusions

Our study reports one of the first cases of successful management of a patient with CCF using the da Vinci® Xi Surgical System. This is a rare case of robotic management of CCF. Preoperative diagnosis of this condition is uncommon and requires the use of various imaging modalities and careful analysis of the clinical presentation. The management of CCF has evolved with surgical technology. We have shown that CCF can be safely and effectively managed with robotic techniques.
